# Investigation of Termite Attack on Cultural Heritage Buildings: A Case Study in Aceh Province, Indonesia

**DOI:** 10.3390/insects11060385

**Published:** 2020-06-22

**Authors:** Novita Novita, Hasbi Amiruddin, Husaini Ibrahim, Teuku Muhammad Jamil, Syaukani Syaukani, Emiko Oguri, Katsuyuki Eguchi

**Affiliations:** 1 Doctoral Program of Social Sciences Education, Universitas Syiah Kuala, Darussalam Banda, Aceh 23111, Indonesia; tm_jamil@unsyiah.ac.id; 2 Department of Family Welfare, Faculty of Teacher Training and Education, Universitas Syiah Kuala, Darussalam Banda, Aceh 23111, Indonesia; 3 Post GraduateProgram, Universitas Islam Negeri Ar-Raniry, Darussalam Banda Aceh 23111, Indonesia; hasbi_amiruddin@yahoo.com; 4 Department of History Education, Faculty of Teacher Training and Education, Universitas Syiah Kuala, Darussalam Banda, Aceh 23111, Indonesia; husib@unsyiah.ac.id; 5 Biology Department, Faculty of Mathematics and Natural Sciences, Universitas Syiah Kuala, Darussalam Banda, Aceh 23111, Indonesia; syaukani@unsyiah.ac.id; 6 Faculty of Education, Tokyo Gakugei University 4-1-1 Nukuikita-machi, Koganei-shi, Tokyo 184-8501, Japan; eoguri@u-gakugei.ac.jp; 7 Systematic Zoology Laboratory, Department of Biological Science, Graduate School of Science, Tokyo Metropolitan University 1-1 Minami Osawa, Hachioji-shi, Tokyo 192-0397, Japan; antist@tmu.ac.jp

**Keywords:** traditional house, wood pest, *Coptotermes*, *Nasutitermes*, Sumatra, tropical Asia

## Abstract

Surveys of the conditions of termite attack were conducted in two regencies, Pidie and Greater Aceh, Aceh Province, Indonesia (40 houses in each location). Interviews were also conducted with home owners to collect data on the building history; culture, such as daily life in the house; the frequency and intensity of termite attacks; and traditional knowledge for avoiding and/or suppressing termite attacks. We found that 51% of traditional houses were infested by two termite species: *Coptotermes gestroi* and *Nasutitermes matangensis*. The lower parts of traditional houses were frequently attacked and severely damaged by termites. Previous land use and the ages of the traditional houses affected the intensity of the termite attacks. Several measures for avoiding and/or suppressing termite attacks on cultural heritage buildings are also proposed.

## 1. Introduction

Various factors can individually or collectively decrease the sturdiness and durability of buildings, as well as their quality and value. Physical factors such as, heat, moisture, and sunlight (UV), may cause the shrinkage, deformation, and degradation of building materials. Furthermore, wood and other materials used in buildings can be destroyed or decomposed by the activities of organisms like termites [1,2] and fungi [3,4]. Damage to buildings is exacerbated by hot and humid climates with a higher level of rainfall and higher activity of wood-eating insect pests [5], especially in tropical areas such as Indonesia. Termites are one of the main types of destructive insects attacking buildings in tropical areas [1,6,7]. 

**Figure 1:**
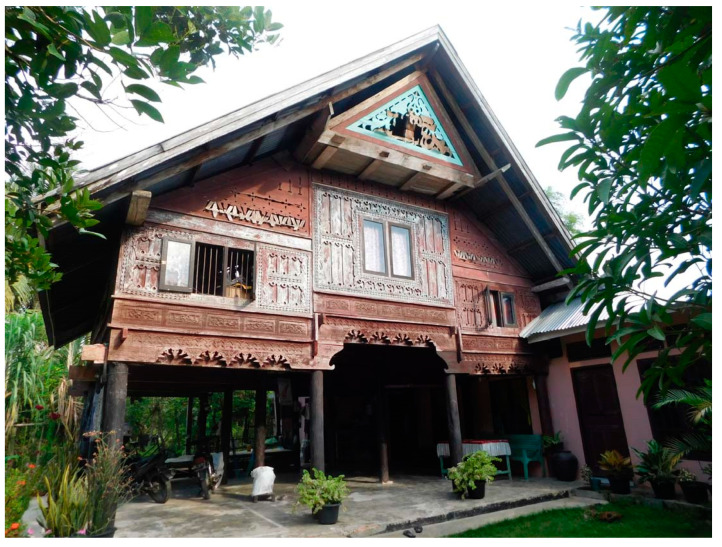
Traditional Acehnese house (stilt-style, with a single story) in Pidie Regency, Aceh, Indonesia. Lower right: semi-modern additional construction (combination of concrete and wood material; pink-wall), not part of the traditional house.

Traditional Acehnese houses are sacred to the social lives of the Acehnese people (Figure 1), an ethnic group from Aceh Province, Indonesia [8]. These houses are decorated with many artistic carvings that symbolize the culture of the people and enriching their daily lives [9,10,11,12]. Traditional houses have irreplaceable value and must be preserved as a cultural heritage for the nation [13,14]. However, most of the existing traditional houses in Aceh suffer from serious damage caused by various factors, including termite attack. Damage to these traditional houses can cause serious economic loss and socio-cultural impacts in Aceh, because such houses are the core of eco-cultural tourism [15,16]. Similar situations are common across the country [12,14,17]. Several species of termite, including *Coptotermes gestroi* (Wasmann) and *Nasutitermes matangensis* (Haviland), are common to the local villages and surrounding forests in Aceh; these termites are suspected to be a potential pest in traditional houses and other wood products.

The subterranean termite, *C. gestroi*, is a serious pest species worldwide [2]. Formerly restricted to Southeast Asia [18], this species’ range has expanded due to the globalization of human activity [19]. This species reached the North American Continent [19] and has become an invasive termite species in Florida (USA), where it attacks native trees, such as Slash pine, *Pinus elliottii* Engelm (Pinaceae), and wood in service [20]. The timber and construction industries are suspected to be the most effective media for the spread of *C. gestroi* in Indonesia. Even though wood preservation methodsagainst *C. gestroi* are well developed [21,22], economic loss caused by this species is increasing in Southeast Asia [21], and *Coptotermes* spp. are known to be serious pests, attacking buildings in China [23], Malaysia [24], and Indonesia [25]. *Coptotermes* is the most economically important genus worldwide [2].

The arboreal-nesting, wood-feeding termite *N. matangensis* is dominant in Sumatran tropical forests. This species, along with other “nasute-group” species, is the main mechanical decomposer of wood. Although *Nasutitermes* spp. are rarely reported to be destructive pests of wood in service (when compared to subterranean termite species, such as *Coptotermes* spp. in Southeast Asia [26,27]), this genus exhibits a relatively wide adaptation to environmental conditions and was reported to be a successful pioneering insect species in the recolonization of the Krakatau Islands (Indonesia) after the catastrophic eruption in 1883 [28,29].

This research aims to investigate and highlight the conditions of termite attack against selected traditional houses in Aceh. The results will be particularly valuable for establishing measures to protect traditional Acehnese houses. The impact of previous land use in relation to termite attack, the ages of the houses, termite attack strategies, and several strategies for suppressing termite attack on traditional houses are discussed.

## 2. Material and Methods

### 2.1. Study Sites

Aceh Province is located in the northernmost part of Sumatra. The area of the province is 56.770 km^2^. Of this, 22,910 km^2^ is forest, 8004 km^2^ is plantation, and 39.28 km^2^ is human developments. This province consists of 23 districts, with a population of more than 5.28 million. This region is defined as tropical, with an annual average temperature of 25.7–28.9 °C [30].

**Figure 2:**
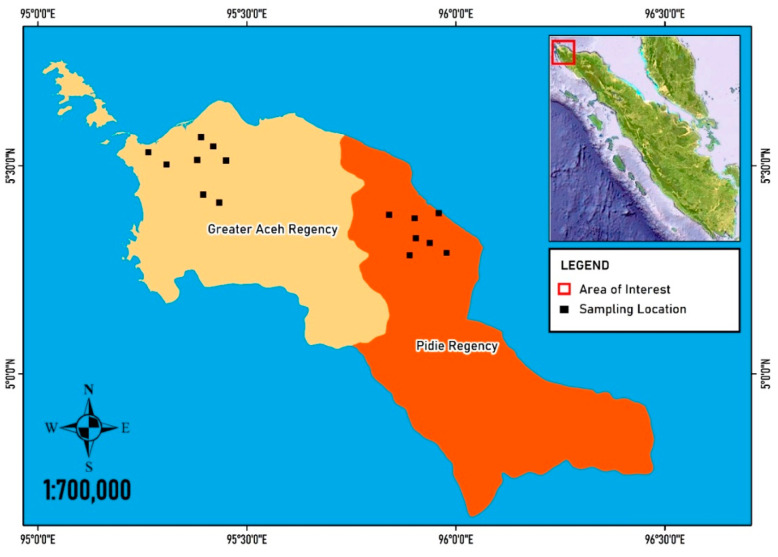
Map of the study sites in the Greater Aceh Regency (pale-yellow) and the Pidie Regency (orange), Aceh Province, Sumatra, Indonesia.

Surveys of termite attack were conducted from March 2018 to July 2019. A total of 80 traditional houses were randomly assessed for termite infestation based on visual observations in the traditional houses preserved in Aceh the Pidie Regency (5°22′13.487″ N 95°56′8.473″ E) (40 houses) and the Greater Aceh Regency 5°27′10.501″ N 95°28′40.012″ E (40 houses), Aceh Province, northern Sumatra, Indonesia (Figure 2). The structure of the houses surveyed was stilt-style, with a single story.

### 2.2. Collection of Termites and Determination of Species

The indications of termite attacks recorded in the present study included galleries (trails of termites covered by soil, wood particles, and/or feces), termite nests around houses, and parts of termite nests that were connected to the buildings. These signs were used to locate and indicate termite attack in the selected structures [31,32,33]. When a traditional house was found to be infested by termites, the condition was recorded digitally and manually. The termites attacking each house were collected in a vial with 70% ethanol and labeled with the house ID number and relevant data. Interviews (semi-structured questionnaires) were also conducted with the home owners to collect data on the building history, culture (such as daily life in the house), the frequency and intensity of termite attacks, and their traditional knowledge of avoiding and/or suppressing termite attacks. Termites were identified by referring to [18,19,34,35,36]. Photographs were taken as multi-layer montages using a Leica M205C stereomicroscope at the Fort Lauderdale Research and Education Center, University of Florida, USA. Collected voucher specimens(preserved in 70% ethanol) are housed in the termite collection of the Biology Department, Faculty of Mathematics and Natural Sciences, Universitas Syiah Kuala.

## 3. Results and Discussion

### 3.1. Termite Species Attacking Traditional Acehnese Houses

Forty-one houses (51%) were attacked by two termite species: *Coptotermes gestroi* and *Nasutitermes matangensis*. 

**Figure 3:**
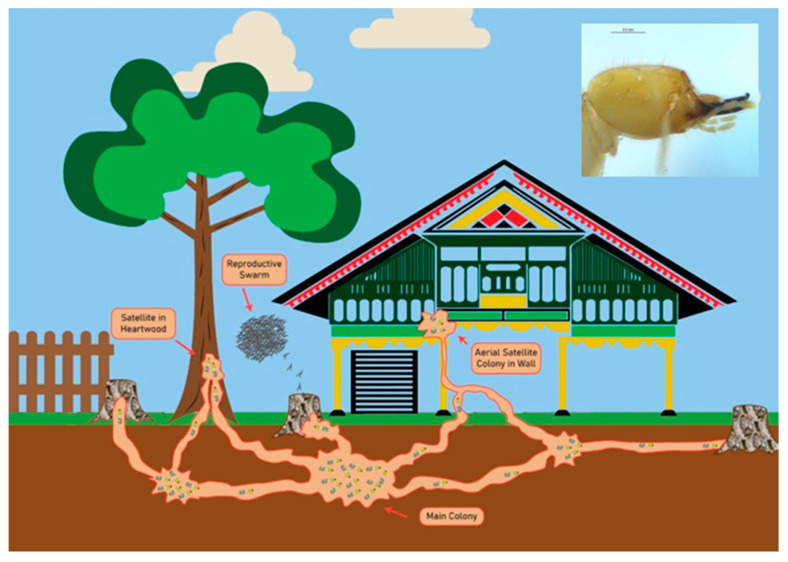
Schematic diagram of *Coptotermes gestroi*’s (top right) strategies of attack against a traditional house [33,34]. Termites from the main nest first attack the lower part of the house through the pillars. The decayed stumps, trees, and wood debris remaining on or under the ground around the houses act as the pathways through which termites attack the house.

Termite attacks caused by other termite species were not found in the present study. We found that *C. gestroi* was the most prevalent destructive termite in the houses surveyed. This subterranean termite species is a common pest in houses in Indonesia, as it attacks wooden structures in both rural and urban areas. Worker and soldier castes invade traditional buildings through wood or other structural parts in contact with or near the ground. The houses with an abundance of wood debris nearby appeared to have higher levels of termite infestation (Figure 3). Infestation by these species was recognized by the presence of mud tubes on buildings up to the roofs (3–4 m), which were often not observed by the occupants.

**Figure 4:**
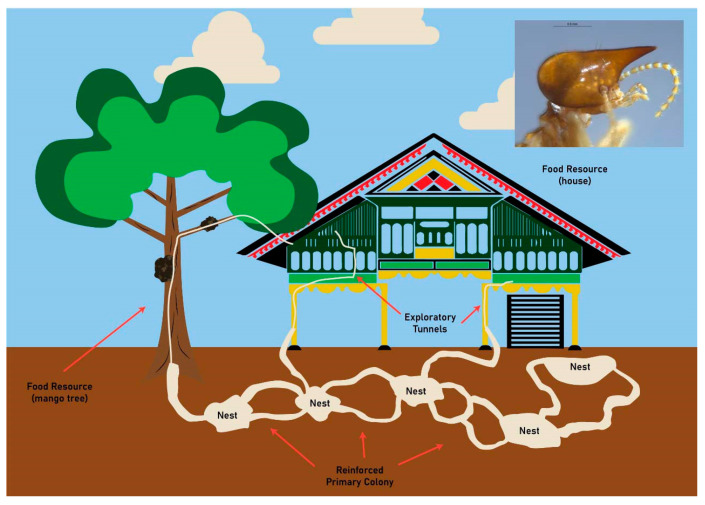
Schematic diagram of *Nasutitermes matangensis*’s (top right) strategies of attack against a traditional house in Greater Aceh Regency. Termites invade from underground and arboreal nests into houses by constructing galleries on branches that come into contact with the house. The branches of the trees around such houses should be regularly trimmed to suppress termite invasion.

Our observations suggest that *N. matangensis* often build their arboreal nests on fruit trees, such as mango (*Mangifera indica* L.) and sapodilla (*Manilkara zapota* L.), surrounding traditional houses and can invade the houses by constructing galleries on branches that come in contact with the house (Figure 4). Abundant and well-functioning galleries indicated a higher activity of termite colonies. *Nasutitermes matangensis* will build several satellite nests in the top corners of houses in the late stages of infestation. The outer cover of the nests is carton-like, soft, and friable, so a nest can be easily broken and penetrated.

### 3.2. Structural Damage to Traditional Houses

**Figure 5:**
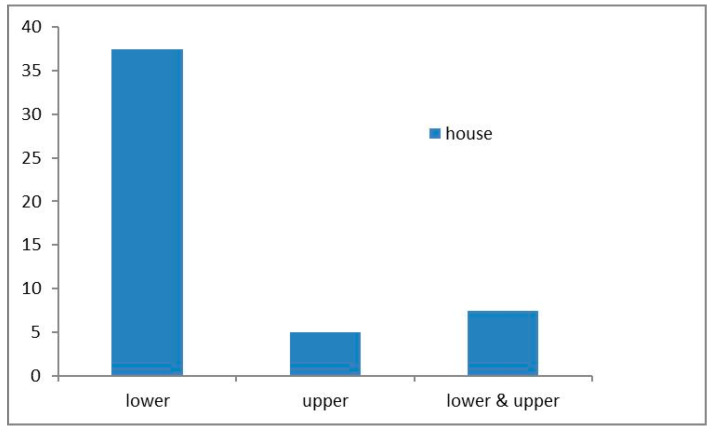
Frequency of structural damage in different parts of the houses surveyed.

**Figure 6:**
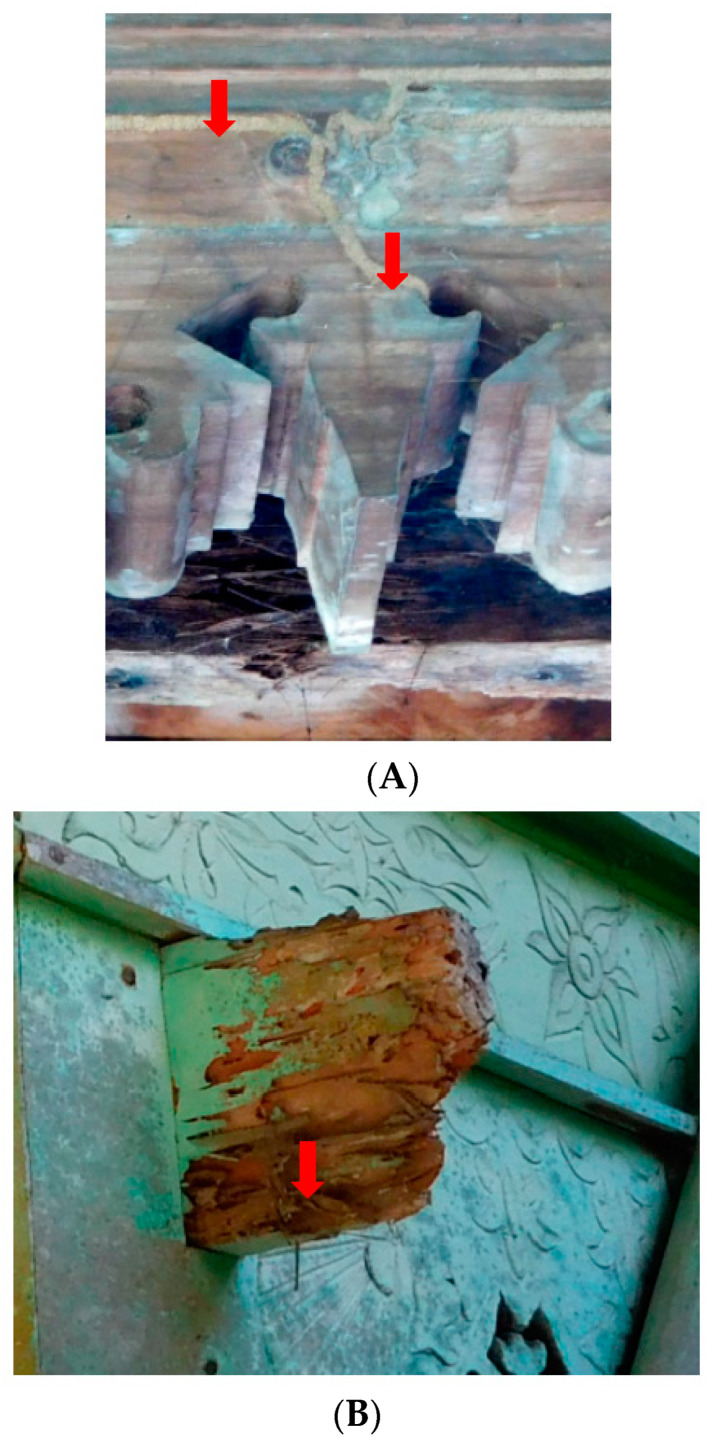
(**A**,**B**) Traditional houses showing moderate termite attack by *Coptotermes gestroi* (Wasmann) in Pidie Regency, Aceh, Indonesia. Red arrows indicate where the termites were collected. A number of galleries ran from subterranean nest(s) to the ornamental parts of the house.

Globally, termites have become a great threat to residential buildings in both rural and urban areas. Termites’ preferences for buildings to attack are related to various factors [37]. We found that the lower parts (pillars, floors, walls, doors, and windows) of traditional houses were frequently attacked (Figure 5) and severely damaged by termites in 31 houses (39% of the 80 surveyed houses), significantly reducing the robustness of the houses (Figure 6). Similar trends were reported in Pakistan [31] and Nigeria [33]. Among the lower parts, pillars tend to be attacked first by *C. gestroi*; this may be due to the pillars usually being in contact with the ground. Even in cases where a stone or concrete foundation had been set at the base under the pillars, termites had constructed galleries over the stone to the wood. Attacks extended to the floors, doors, walls (ornaments), and roof. Conversely, the upper house parts (roofs and gables) showed attacks in four of the houses (5%) surveyed, and both the lower and upper parts were attacked in six of the houses (8%) surveyed.

**Figure 7:**
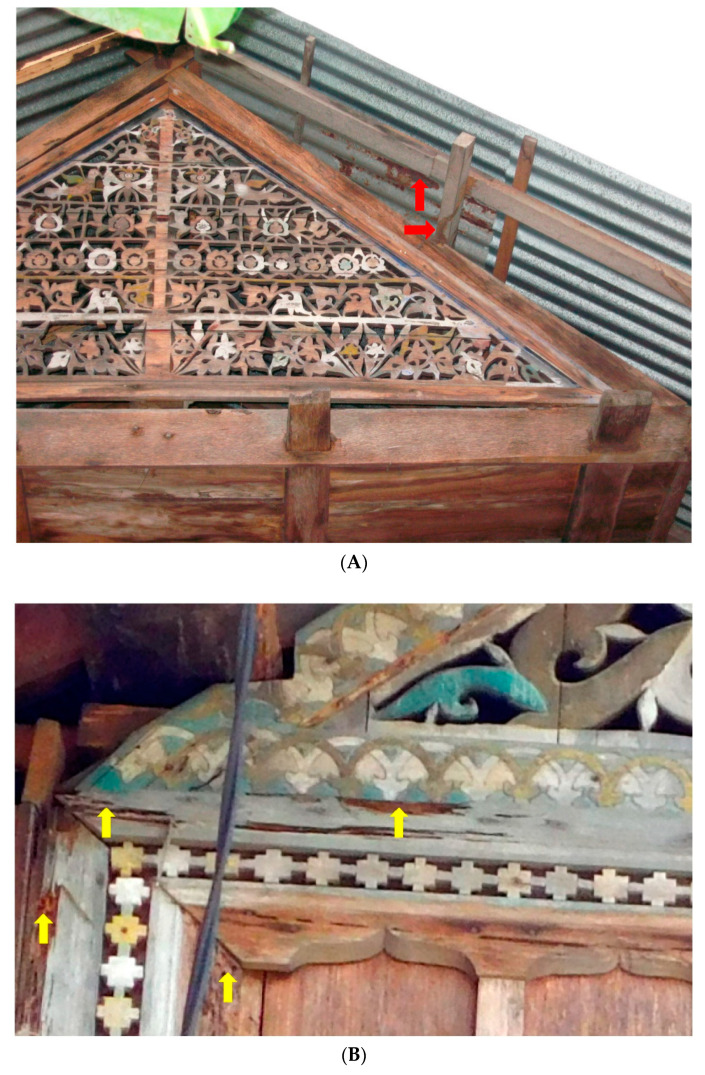
(**A**,**B**) Traditional houses attacked by *Nasutitermes matangensis* (Haviland) in the Greater Aceh Regency. Red arrows indicate where the termites were collected. A number of large galleries ran from arboreal and underground nests. Workers actively carried wood particles back to the nests through the galleries (**A**). The ornamental parts (indicated by a yellow arrow) and walls were heavily attacked by the termites (**B**). Both of the houses were unoccupied.

*Coptotermes gestroi* build their nests in the soil, and workers forage away from the nest to find wood that generally has a high moisture content. This species usually first consumes the wood inside walls and poles and builds pathways (thus, the main structures of the infested houses seemed to remain intact when seen from the outside); they then reach other parts of the house using the inner pathways, as well as galleries. We found that *N. matangensis* usually consumes the outer parts of house wooden structures first (Figure 7). 

We also found that termite attack in the lower and upper parts of the surveyed houses may have been associated with the presence of fungal decay. We suspect that fungi accelerate the termite’s ability to attack wood by reducing wood density, as it is has been shown that wood consumption by termites can be correlated to the degree of wood decay [38,39]. For example, *Coptotermes acisnaciformis* (Froggatt) is attracted to and consumes wood decayed by brown and white rot fungi [40]. The odor of fungal mycelium also appears to stimulate termite activity [4,41]. Poor ventilation, damp conditions, and poor cleanliness in and around the house likely accelerate termite attacks coupled with fungal decay.

### 3.3. Previous Land-Use

**Figure 8:**
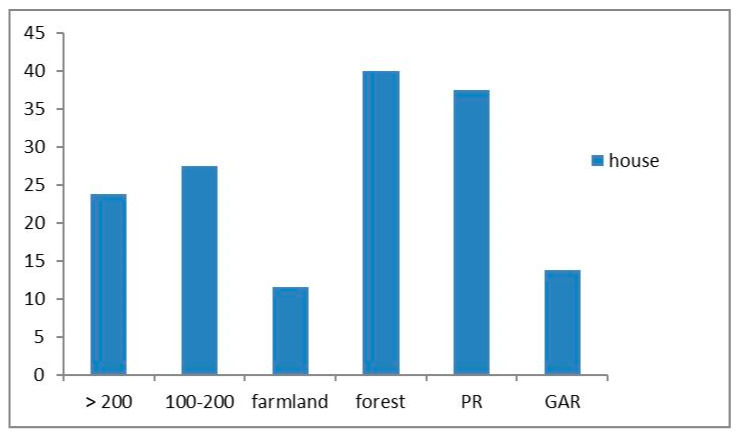
Frequency of termite attack in traditional houses for different house ages (>200 or 100–200 (years)), previous land use (farmland or forest), regencies, and survey sites (Pidie Regency (PD) or Greater Aceh Regency (GAR)).

All the traditional houses that we surveyed were located in a previously forested area that had been logged and cleared for farmland. Previous land use might be related to the frequency and intensity of termite attacks on traditional houses. Termite attack was recognized in 31 traditional houses (78% of 41 attacked houses) built in areas that had experienced forest logging. We observed termite attack in 11 houses (22%) built in areas transformed from farmland (Figure 8). We also found that a number of rotten stumps still remained on or under the ground around the some of the surveyed houses. These are a major risk factor for termite attack, because they can become nesting sites for *C. gestroi* from which the termite attack can expand to the nearby houses (Figure 3). Mo et al. [42] reported that subterranean termites like *Coptotermes* spp. are predominantly found in housing areas that were previously covered by forest or used as farmland. The wood debris mixed into the soil after land clearing becomes a food source and nesting site for subterranean termites [27].

### 3.4. Age of Traditional Houses

According to interviews with home owners, 72 houses (90%) were built using selected hard wood species (e.g., *Shorea* spp., *Artocarpus* spp., and *Vitex* spp.) depending on their culture, art, beliefs, and traditional knowledge of biological resources, as reported in Saudi Arabia [9], Malay Peninsula [10], and Africa [32]. Such wood species were common around the villages at the time of construction. However, with increasing deforestation, these wood species have become scarce and expensive. The local people have been forced to use low-quality wood for building or repairing their houses. These circumstances make such houses vulnerable to termite disturbances, as well as other physical disturbances, such as storms and earthquakes.

Termites usually prefer soft woods compared to hard woods [43] because the latter contains a larger amount of lignin and is not easily digested by termites [1,44]. Undigested lignin is excreted by termites as feces and used for nest building [45]. Traditional Acehnese houses were usually made with preferred hardwood species, often with a combination of wood and concrete. We found that the ages of traditional houses affected the intensity of the termite attacks. All the traditional houses that were more than 200 years old (19 houses) were attacked by termites at a serious (32%) and moderate level (68%) (Figure 8). Many wood species used for traditional houses contain chemical compounds that can suppress the attacks of xylophageous insects [46]. Some wood species are preferred by termite species [47]. However, given the long time since their construction (>200 years), the quality of the wood had deteriorated due to environmental factors. Basidiomycete fungi can change the wood’s physical–mechanical properties and reduce the durability of wooden materials [48] that are preferred by termites [22]. Fungal mycelium can trigger and accelerate termite activity and consequently increase the level of wood destruction [4,49]. The annual local climate conditions in Aceh (24–29 °C and 80% humidity) are ideal for fungal growth and termite activity.

## 4. Conclusions

All the traditional houses that we surveyed were located in areas developed after forest logging or transformed from farmland that remained close to farmland or the edges of forests. These conditions appeared to help both subterranean and arboreal termites to infest houses. A lack of cleanliness around the houses and home age (100 to >200 years old) were also found to be risk factors for termite attack. In recent years, it has been difficult to obtain durable wood. Consequently, homeowners tend to use lower quality wood to repair their houses. This might enhance termite attack against traditional historic houses. The frequency and intensity of termite attacks seem to have rapidly increased in the last 20 years. The activities of the timber, construction, and manufacturing industries, which use wood on large geographic scales, may have exacerbated the recent termite situation.

We recommend the following measures for suppressing termite attack against cultural heritage houses: (1) remove decayed wood debris and unnecessary wood products on and under the ground around houses, (2) regularly trim the branches of trees around houses, (3) provide good quality wood for repairing houses, and (4) regularly monitor for signs of termite attack. It is necessary to pay attention to the activities of the timber, construction, and manufacturing industries. By contrast, we do not recommend the use of highly toxic insecticides in land [50]. Traditional houses also provide potential nesting sites and nest-building materials for bees and wasps. It is widely known that bees are important pollinators of crops and that wasps act as effective natural enemies of crop pests in rural agro-ecosystems [50,51]. The baiting protocol suggested by Su et al. [52] could be adjusted and used in this area of cultural heritage buildings in Aceh and other parts of Indonesia where these termite species are prevalent.
